# Interband Cascade Active Region with Ultra-Broad Gain in the Mid-Infrared Range

**DOI:** 10.3390/ma14051112

**Published:** 2021-02-27

**Authors:** Krzysztof Ryczko, Agata Zielińska, Grzegorz Sęk

**Affiliations:** Department of Experimental Physics, Faculty of Fundamental Problems of Technology, Wrocław University of Science and Technology, Wybrzeże Wyspiańskiego 27, 50-370 Wrocław, Poland; 236442@student.pwr.edu.pl (A.Z.); grzegorz.sek@pwr.edu.pl (G.S.)

**Keywords:** interband cascade laser, optical gain, quantum well, mid-infrared range

## Abstract

The optical gain spectrum has been investigated theoretically for various designs of active region based on InAs/GaInSb quantum wells—i.e., a type II material system employable in interband cascade lasers (ICLs) or optical amplifiers operating in the mid-infrared spectral range. The electronic properties and optical responses have been calculated using the eight-band k·p theory, including strain and external electric fields, to simulate the realistic conditions occurring in operational devices. The results show that intentionally introducing a slight nonuniformity between two subsequent stages of a cascaded device via the properly engineered modification of the type II quantum wells of the active area offers the possibility to significantly broaden the gain function. A −3 dB gain width of 1 µm can be reached in the 3–5 µm range, which is almost an order of magnitude larger than that of any previously reported ICLs. This is a property strongly demanded in many gas-sensing or free-space communication applications, and it opens a way for a new generation of devices in the mid-infrared range, such as broadly tunable single-mode lasers, mode-locked lasers for laser-based spectrometers, and optical amplifiers or superluminescent diodes which do not exist beyond 3 µm yet.

## 1. Introduction

An important class of semiconductor lasers operating efficiently in the spectral range of the first atmospheric window (2.9–5.3 μm), coinciding with the strongest absorption lines of many environmentally relevant gasses, utilizes a cascaded scheme of interband transitions in type II quantum well (QW) structures [1,2,3,4]. Hence, such devices are called interband cascade lasers (ICLs) and they are usually based on a broken gap material system of InAs/GaInSb [2,3,5,6]. The usage of such materials, besides the natural band edge alignment which is necessary in ICLs, allows the emission in a broad range of the mid-infrared (MIR) [7] and the growth on GaSb or InAs substrates to remain in a relatively low strain limit range. This assures the high structural and optical quality of epitaxially grown III-V materials and hence a good device performance. All these properties cannot be obtained in other material systems suitable for the MIR. When, for instance, diode lasers (based on type I optical transition in GaSb-based quantum wells) are considered, it becomes more and more difficult to reach the threshold with increasing emission wavelength due to the lower gain. Therefore, multiple quantum wells are used to ensure sufficient amplification while maintaining reasonable threshold current densities. This in turn leads to significant proportion of the parasitic voltage drops in the passive areas of the device in the total voltage. Thus, the threshold power density increases. In addition, with the increased emission wavelength (decreased optical transition energy) the carrier losses and probability of nonradiative processes as the Auger recombination increase [6,8]. All these problems can be overcome or minimized by employing a cascaded scheme, which was proposed to be used in interband cascade lasers [5,9]. These lasers can operate from 2.9 to 7 μm at least (where the longer wavelength results concern structures on InAs substrates) [10,11,12,13], with threshold drive powers 1–2 orders of magnitude lower than for quantum cascade lasers (QCLs) [11], while still generating 500 mW of continuous wave (cw) output power [12]. Therefore, GaSb-based ICLs are ideal for MIR spectroscopy and gas sensing applications that rarely require Watt-level optical power [13].

The success of ICLs partly comes from a freedom in their band structure and optical properties engineering. This is achievable due to the spatially indirect character of the active optical transition and the possibility of adjusting the type-II QW layers’ thicknesses or compositions in a broad range [14,15,16,17]. This, in turn, allows tailoring the strain, band gap discontinuities, optical transition energy (wavelength), or valence band states intermixing and hence the polarization of the emitted or absorbed light [18,19].

Recent developments have made possible the fabrication of ICLs with record low power consumption (<0.1 W at threshold at 300 K) and ultralow threshold current densities as for MIR lasers [12,20], as well as high cw output powers [3], continuous wave and single-mode operation at room temperature or even above, in the spectral range from below 3 to above 6 μm [20,21,22,23,24]. As a result, the performance of ICLs has met the requirements of a variety of applications already, such as optical gas sensing [25], industrial process control [26,27], environmental pollution monitoring [28], medical diagnostics [29], infrared countermeasures [30], gas leakage detection [31] and free space optical communication [32]. In some of these applications, the broad gain function of the active material is especially advantageous. For instance, it is important when aiming at wide spectral tunability of the laser emission but also essential in the context of mode-locked lasers and tailoring their pulse duration. Eventually, spectrally broad gain becomes critically necessary for semiconductor optical amplifiers or superluminescent diodes in the MIR. Such broad gain solutions are basically still missing in the spectral range beyond 3 μm, therefore this work attempts to fill this gap and paves the way towards new generation of devices, utilizing however the knowledge and experience from previous developments in ICLs.

In this paper, we discuss the design strategies allowing for extraordinary spectrally broad gain active material in the range of about 3–5 μm. It is shown that the optical gain function in the InAs/GaInSb structures can be controlled by an appropriate number of wells and their thicknesses, as well as the effective overlap of the subsequent absorption response leading to the significant extension of the resulting gain.

## 2. Materials and Methods

To calculate the electronic structure of type II W-design active region—i.e., utilizing coupled InAs/GaInSb QWs—we employ the k·p theory and use 8 × 8 Hamiltonian defined for the [001] growth direction. Our eight-band model includes also the strain effects after Ref.14 for two different types of substrate materials—InAs or GaSb. External electric field has also been taken into account to simulate the conditions occuring during ICLs operation. The carrier wave functions and the subband energies are determined by numerically solving the Schrödinger equation employing the finite difference method (FDM). The FDM is suitable for this kind of calculations because it is fast and facilitates using an arbitrary mesh [33]. To calculate the electronic subband structure and wave function we use the standard mathematical subroutines available in the LINPACK libraries [34]. More details on the calculations’ methodology can be found in Reference [14]. All the material parameters were taken from Reference [35] for 300 K. The parameters for the ternary compounds are obtained using a linear interpolation of the binary materials’ parameters. In the case of the Ga_x_In_1−x_Sb band gap, the bowing parameter is included (i.e., Eg(Ga_0.76_In_0.24_Sb) = 0.518 eV). The most important ones are gathered in Table 1 below.

After obtaining the band structure and the wave functions, the next step is to calculate the optical gain, as one of the key parameters to evaluate the performance of a device. Using Fermi’s golden rule, the optical gain of a quantum well for the transition between the initial (*m*th conduction subband) and final (*n*th valence subband) states can be expressed as [36]:

(1) $$g_{mn}\left(E\right)=\frac{\pi e^{2}\hbar }{n_{r}c\varepsilon_{o}m_{o}^{2}LE}\;\int {\left|\hat{e}\cdot M_{mn}\left(k_{\left|\right|}\right)\right|}^{2}\times \left(f_{m}^{e}\left(E\right)-f_{n}^{v}\left(E\right)\right)\delta \left(E_{m}^{e}\left(k_{\left|\right|}\right)-E_{n}^{v}\left(k_{\left|\right|}\right)-E\right)dk_{\left|\right|}$$

where $f_{m}^{e}\left(E\right)\;$and $f_{n}^{v}\left(E\right)$ are the Fermi distribution functions for electrons and holes, respectively; e is the elementary electric charge; ℏ is the Planck constant; $n_{r}$ is the refractive index; c is the speed of light; $\varepsilon_{o}$ is the dielectric constant of vacuum; $m_{o}\;$is the free electron mass; *L*—width of the quantum wells; *E* is the photon energy; $\hat{e}$ is a unit vector along the polarization direction of the optical field. $M_{mn}\left(k_{\left|\right|}\right)$ is the momentum matrix element between the *m*th conduction subband and the *n*th valence subband. The momentum matrix elements are evaluated by using the Bloch states for electrons and holes.

The optical transition matrix elements between the valence (hole) subbands and the conduction (electron) subbands are given by:

(2) $$M_{i}^{c,\;v}=\langle \Psi_{c}|\hat{e}\cdot {\hat{p}}_{i}{|\Psi }_{v}\rangle ,\;i\;=\;x,\;y,\;z$$

where $\hat{e}$ is the unit vector of the electric field direction; ${\hat{p}}_{i}$ is the momentum operator; and $\Psi_{c}$ and $\Psi_{v}$ are the electron and hole wave functions, respectively. The electron (hole) wave function in a QW is given by:

(3) $$\Psi_{c\left(v\right)}\left(\vec{r}\right)=Ae^{i{\vec{k}}_{\left|\right|}\cdot {\vec{r}}_{\left|\right|}}\sum_{j=1}^{8}\phi_{j}\left(z\right)|u_{j}\left(\vec{r}\right)>$$

where $|u_{j}\left(\vec{r}\right)>$ are the Bloch wave functions at the center of the Brillouin zone. We have used the 8 × 8 k·p Hamiltonian matrix for zinc-blende semiconductor with the following basis:

(4) $$|u_{1}\rangle =i|S\;↑\rangle ,\;\Gamma_{6}\;|u_{2}\rangle =i|S\;↓\rangle ,\;\Gamma_{6}\;|u_{3}\rangle =\frac{i}{\sqrt{2}}(|X\;↑\rangle +i|Y\;↑\rangle ),\;\Gamma_{8}\;|u_{4}\rangle =\frac{i}{\sqrt{6}}\left(|X\;↓\rangle +i|Y\;↓\rangle \right)-i\sqrt{\frac{2}{3}}|Z\;↑\rangle ,\;\Gamma_{8}\;|u_{5}\rangle =\frac{i}{\sqrt{6}}\left(|X\;↑\rangle -i|Y\;↑\rangle \right)+\sqrt{\frac{2}{3}}|Z\;↓\rangle ,\;\Gamma_{8}\;|u_{6}\rangle =\frac{i}{\sqrt{2}}(|X\;↓\rangle -i|Y\;↓\rangle ),\;\Gamma_{8}\;|u_{7}\rangle =\frac{i}{\sqrt{3}}\left(|X\;↓\rangle +i|Y\;↓\rangle \right)+|Z\;↑\rangle ,\;\Gamma_{7}\;|u_{8}\rangle =-\frac{i}{\sqrt{3}}\left(|X\;↑\rangle -i|Y\;↑\rangle \right)-|Z\;↓\rangle ,\;\Gamma_{7}$$

where |S〉 denotes the *s*-like conduction band Bloch state and |X〉, |Y〉, and |Z〉 denote the *p*-like valence band Bloch states. Substituting Equations (3) and (4) into (1) we obtain the squared optical matrix elements (for a QW grown along z direction), M_x_ or M_y_ correspond to the transverse electric (TE) modes of optical transition of interest, as these are the ones constituting output of the ICL (calculation methodology after ref [37]). Using a specific electron density *n*, and charge neutrality condition *n* = *p*, where p is the holes’ density, we found the quasi-Fermi levels by integrating the density of states multiplied by the occupation probability over the entire band. For electrons (n) and for holes (p), the respective densities are given by [37]:

(5) $$n=\sum_{m}\int \rho_{m}^{e}\left(E\right)f^{e}\left(E_{m}^{e}\left(k_{\left|\right|}\right)\right)dE$$

and

(6) $$p=\sum_{n}\int \rho_{n}^{h}\left(E\right)(1-f^{h}\left(E_{n}^{h}\left(k_{\left|\right|}\right)\right)dE$$

Here, $\rho_{n}^{e\left(h\right)}\left(E\right)$ is the 2D density of states for electrons (holes). The total gain is then obtained by using the subband-to-subband optical gain and is given by [36,38]:

(7) $$g\left(E\right)=\int g_{mn}\left(E-E^{\prime }\right)ℒ\left(E^{\prime }\right)dE^{\prime }$$

where $ℒ\left(E^{\prime }\right)$ is a Lorentzian line-broadening function [36,38]:

(8) $$ℒ\left(E^{\prime }\right)=\;\frac{1}{\pi }\;\frac{\Gamma }{{\left(E-E^{\prime }\right)}^{2\;}+\;\Gamma^{2}}$$

where Γ is the half linewidth of the Lorentzian function. We estimate Γ to be equal to 5 meV, based on the transport data for *n*- and *p*-type InAs/Ga_1−x_In_x_Sb superlattices of type II [39]. Below, we present the results for TE polarization only.

## 3. Results and Discussion

We first consider the influence of various modifications in an ICL-like active region on the fundamental optical transition energy. An important point which cannot be neglected and occurs in most operational devices is the biasing of the structure with an external voltage and hence the existence of an electric field in the active region.

**Figure 1:**
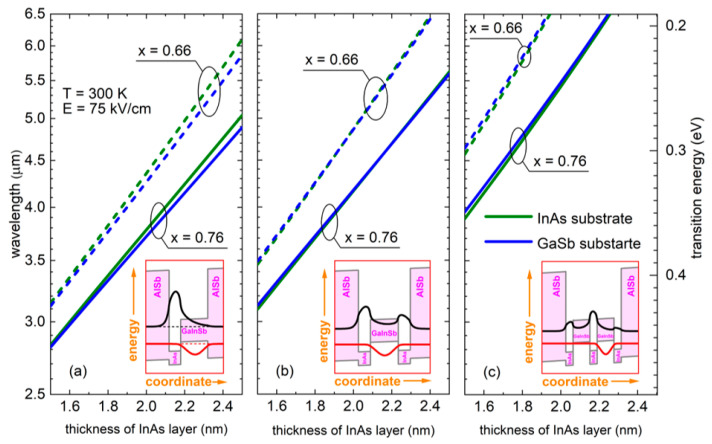
Fundamental transition energy in the active region of AlSb/InAs/Ga_x_In_1−x_Sb/AlSb (**a**), AlSb/InAs/Ga_x_In_1−x_Sb/InAs/AlSb (**b**) and AlSb/InAs/Ga_x_In_1−x_Sb/InAs/Ga_x_In_1−x_Sb/InAs/AlSb (**c**), as a function of the InAs well width. Two different values of the Ga content in Ga_x_In_1−x_Sb are used: 66% (dashed line) and 76% (solid line). Blue and green lines refer to structures deposited on GaSb and on InAs, respectively. The Ga_x_In_1−x_Sb layer thickness is 3.5 nm. The insets show the confinement potential profiles, energy levels and carrier probability densities in the external electric field.

Figure 1a–c present the calculated fundamental transition energy dependence on the InAs layer thickness. We consider a single-stage active region formed of AlSb/InAs/Ga_x_In_1−x_Sb/AlSb (one InAs layer), AlSb/InAs/Ga_x_In_1−x_Sb/InAs/AlSb (two InAs layers—the so called W-design), AlSb/InAs/Ga_x_In_1−x_Sb/InAs/Ga_x_In_1−x_Sb/InAs/AlSb (triple InAs QW). In this first step, the input data have been taken as those typically used in ICLs—i.e., Ga contents of 66% or 76%, Ga_x_In_1−x_Sb layer of 3.5 nm width, and an electric field of 75 kV/cm [40]. What is mainly seen in Figure 1 is the possibility to cover the target range in the MIR of at least 3–5 µm through just changing the InAs layer thickness, which is an approach very often used in ICLs [14,41]. We intentionally limited ourselves to the values around 0.66–0.76 of gallium. The reason for this is very practical—from the growth point of view, it is necessary to remain below certain strain limit to assure coherent layer growth and to avoid risk of reaching the critical thickness, therefore lower Ga contents (more indium) are excluded. On the other hand, we did not consider larger Ga contents because, in this case, the appropriate adjustment of energy levels becomes difficult and hence large Ga content values are not used in the actual devices (see, e.g., [6]). We use a AlSb/InAs/Ga_x_In_1−x_Sb/InAs/AlSb structure, because such a solution is technologically straightforward as AlSb belongs to the same materials’ family, it is commonly used in fabrication of ICL devices and works as a confinement barrier for both types of carriers, electrons and holes.

The insets in Figure 1a–c present sketches of possible realizations of the active region plus the probability densities of the ground electron and hole states. In such type II structures constituting a broken gap system, the fundamental interband optical transition occurs between electron state localized mostly in the InAs layers and hole state concentrated in the Ga_x_In_1−x_Sb layers. Therefore, in all the cases we are dealing with spatially indirect transitions. External electric field pushes the electron and hole probability distributions further in the opposite directions, which is especially visible in the inset of Figure 1c—i.e., effectively wider confinement potential.

**Figure 2:**
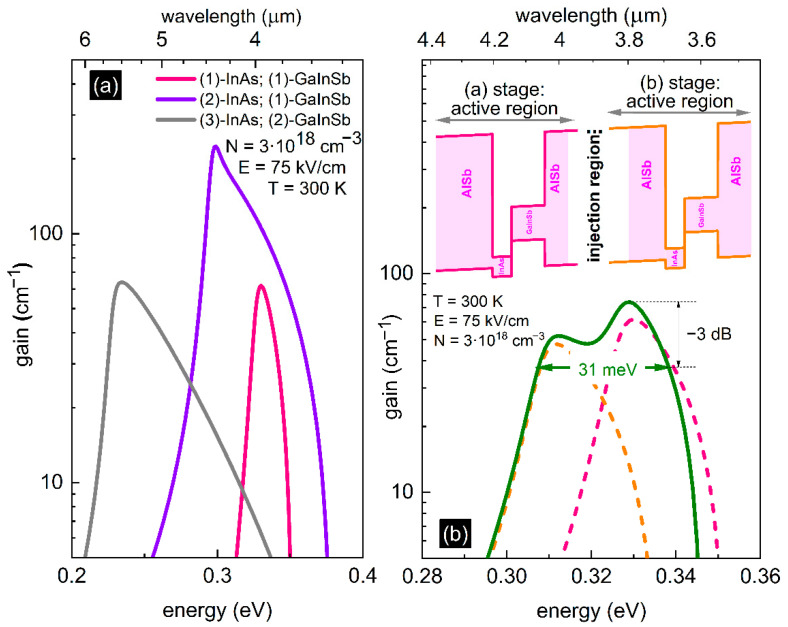
Optical gain spectra: (**a**) for one stage: AlSb/InAs/Ga_x_In_1−x_Sb/AlSb—pink solid line, AlSb/InAs/Ga_x_In_1−x_Sb/InAs/AlSb—violet solid line and AlSb/InAs/Ga_x_In_1−x_Sb/InAs/Ga_x_In_1−x_Sb/InAs/AlSb—gray solid line (x = 0.76; width of InAs layer equal to 2.0 nm); (**b**) for two stages, wherein each individual active region (1 stage) AlSb/InAs (2.0 nm-dashed pink line/2.1 nm-dashed orange line)/Ga_0.76_In_0.24_Sb (3.5 nm)/AlSb; broad combined gain (2 stage design)—solid green line.

In Figure 2a, we present the optical gain for all three designs considered in Figure 1, for a single stage case. We see that maximum values of the optical gain equal about 60 cm^−1^ for structures with one InAs layer and GaInSb layer as well as two InAs layers and three GaInSb layers, but 220 cm^−1^ for two InAs layers and one GaInSb layer. This is caused by the external electric field, which strongly modifies the electron-hole overlap integral.

In Figure 2b, the calculated optical gain spectrum is plotted for an exemplary injected carrier density of 3 × 10^18^ cm^−3^ and electric field of 75 kV/cm (both values are characteristic for ICLs [37,40,42]). The dashed lines correspond to the results for a single QW (1 stage) case for two slightly different wells, with InAs thicknesses equal to 2.0 and 2.1 nm, respectively. The maximum gains are slightly different (62 and 48 cm^−1^, respectively), whereas the −3 dB gain widths are about 220 nm in each of the two cases. To increase the gain bandwidth, we combine two pairs of such slightly different wells inside a two stage active region, where the two QWs are separated by a hypothetical injector region. A scheme of such a structure in external electric field is presented in the inset of Figure 2b. When such an inhomogeneity is intentionally introduced between the stages of a cascaded device, the gain width increases to about 380 nm (i.e., 31 meV in energy scale).

**Figure 3:**
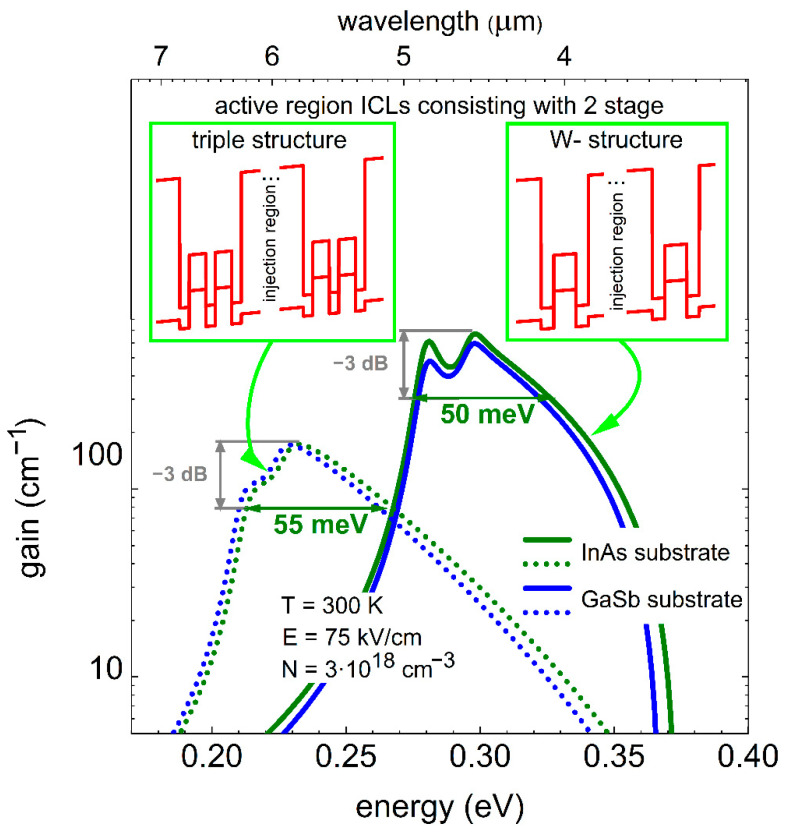
Optical gain functions for different active region consisting of 2 stages with a W-structure (solid line) made of AlSb/InAs (2.0 nm)/Ga_0.76_In_0.24_Sb (3.5 nm)/InAs (2.0 nm)/AlSb and AlSb/InAs (2.1 nm)/Ga_0.76_In_0.24_Sb (3.5 nm)/InAs (2.1 nm)/AlSb and triple structure (dotted line) consisting of AlSb/InAs (2.0 nm)/Ga_0.76_In_0.24_Sb (3.5 nm)/InAs (2.0 nm)/Ga_0.76_In_0.24_Sb (3.5 nm)/InAs (2.0 nm)/AlSb and AlSb/InAs (2.1 nm)/Ga_0.76_In_0.24_Sb (3.5 nm)/InAs (2.1 nm)/Ga_0.76_In_0.24_Sb (3.5 nm)/InAs (2.1 nm)/AlSb. Blue and green lines refer to structures deposited on GaSb and on InAs, respectively.

Figure 3 shows the similarly calculated optical gain spectra for two other two-stage designs, in which the QW thicknesses are also 2 and 2.1 nm, but this time the W-like QW structure made of AlSb/InAs/GaInSb/InAs/AlSb and triple AlSb/InAs/GaInSb/InAs/GaInSb/InAs/AlSb QW (see insets in Figure 3), on two different substrates (different strain conditions) of GaSb and InAs, are considered. The obtained gain widths are approx. 50 meV (850 nm) and 55 meV (1140 nm), respectively—i.e., it is significantly increased when compared to the case presented in Figure 2. This is mostly caused by the effectively wider confinement potential of the type II QWs, which in turn causes larger inhomogeneity in a sense of the electronic levels difference in electric field between the two stages of a device. It is also worth noting that the difference between the cases of two substrates is rather small—the effects are dominated by wide QW potentials. The difference in gain widths between the double and triple inhomogeneous QW designs is only 290 nm in favor of the latter, but sacrificing the gain maximum by 50%. So, in any final operational device there will always be a compromise between broader or higher gain.

Although the triple design structure provides larger bandwidth with however lower gain, it is also more challenging to fabricate—high precision in larger number of very thin QW layers is required. The optical gain spectra and energy peak position are reduced for the triple structure (as compared with W-structure), because triple structure has in fact an effectively wider confinement potential for both electrons and holes. It causes that in the external electric field the wave functions of electrons and holes are separated stronger than in the W-structure and more down-shifted inside the tilted QW potential (basically due to quantum confined Stark effect). This tendency is seen in schemes in Figure 1b,c. Thus, one can suspect that in the real device of such a triple QW design it might be difficult to reach the ultimate performance regarding the gain bandwidth. Therefore, in the next part we limit ourselves only to the W-like active region as more realistic one to achieve the record large gain width. It will also have other advantages, when for instance used in ICL, as higher gain will be beneficial and translate into lower threshold currents in such a case.

**Figure 4:**
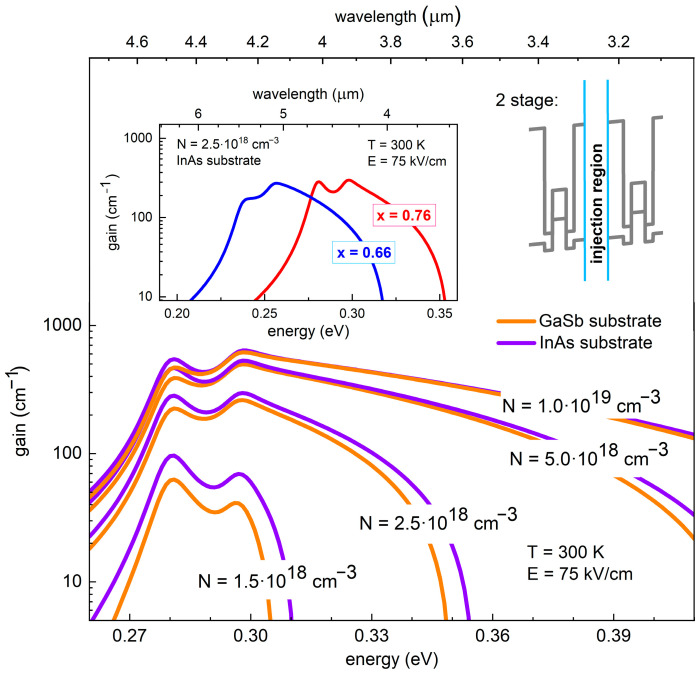
Optical gain functions for two different free carrier concentrations and the following design: 2 stage AlSb/InAs (2.0 nm)/Ga_0.76_In_0.24_Sb (3.5 nm)/InAs (2.0 nm)/AlSb and AlSb/InAs (2.1 nm)/Ga_0.76_In_0.24_Sb (3.5 nm)/InAs (2.1 nm) AlSb. The solid orange and violet lines refer to structures deposited on InAs and on GaSb, respectively. The inset shows comparison of the optical gain for two possible Ga content in Ga_x_In_1−x_Sb (66%—blue and 76%—red solid line, respectively).

Figure 4 shows the broad optical gain as a function of the energy (wavelength) calculated for a 2-stage W-like AlSb/InAs/GaInSb/InAs/AlSb QW and for various, but still realistic, values of the carrier densities in the active region, between 1.5 × 10^18^ cm^−3^ and 1.0 × 10^19^ cm^−3^ [6,11,37]. When following the carrier density dependence, the gain function changes quite dramatically indicating on the importance of this parameter in the final device. It is rather intuitive that increasing the carrier concentration implies higher and broader gain (stronger overall absorption in a broader spectral range of integration). The differences in the obtained results for the cases of the two considered substrates (which is mainly the effect of differences in strain which only slightly affects the confined states) are not significant, however slightly better values of both the gain maximum and the gain width were obtained for InAs substrate. The natural lattice constant of InAs is larger than that of GaSb, therefore, when the layer of InAs is grown on GaSb, the in-plane strain equals 0.62% and the InAs layer is subjected to slight biaxial tensile strain (this strain is absent in the case of the InAs substrate). Whereas, in the case of the GaInSb layers there is some in-plane compressive strain for both substrates, but of slightly different value. For instance, for Ga_0.76_In_0.24_Sb the strain equals −1.51% and −2.14% when grown on GaSb and InAs substrate, respectively, (similarly, for Ga_0.66_In_0.34_Sb the strain equals −2.1% and −2.77% for GaSb and InAs substrate). Therefore, for the case of the GaSb substrate, the electron levels (electrons are mostly localized in the tensile-strained InAs layers) will slightly shift down in the energy scale, and hence the “leakage” of the electron wave function into the GaInSb layer gets smaller. This in turn causes a decrease in the overlap integral between the electron and hole wave functions and hence the lower intensity of the optical transition (absorption). It is the other way around for holes, which partly compensates the effects for electrons but is less significant due to much larger effective masses for holes—both the energy level shift and the change in the spatial distribution of the wave function (probability density) are smaller. This is why the optical gain spectra are very similar for both substrates (differing strain conditions), however with a bit smaller gain for the GaSb case, for which the effect of tensile strain on electron states dominates. For the highest carrier density, the gain maximum exceeds 500 cm^−1^ (almost identical for both substrates), whereas the gain width is a bit larger for the InAs substrate and reaches almost 900 nm.

## 4. Conclusions

We have theoretically studied various designs of ICL active region made of combinations of type II InAs/GaInSb materials in the context of maximizing the width of optical gain spectrum, as demanded by many optoelectronic applications in the mid infrared range. We have also taken into consideration the practical aspects relevant for real devices and included the effects of the substrate, external electric field, and free carrier densities. We have shown that intentionally introduced inhomogeneity between two stages of a cascaded device structure can lead to a significant increase in the gain spectral bandwidth to about 1 μm in the relevant range of at least 3–5 µm, which is almost an order of magnitude larger than that reported for any currently existing ICL. This, in turn, opens a way towards a completely new generation of devices long sought and highly demanded in many applications, requiring broad optical response or wide spectral tunability, especially those related to gas sensing. Therefore, we hope that these results, which are also almost insensitive to the substrate material (GaSb vs. InAs), stimulate the technological groups and bring us closer to the realization of such broad gain devices in the MIR.

## Figures and Tables

**Table 1:** Material parameters for the calculation.

Parameter/Material	Symbol (Unit)	InAs	AlSb	GaSb
Band gap	Eg (eV) (300 K)	0.356	2.300	0.726
Spin-orbit energy	Δ (eV)	0.390	0.676	0.760
Interband matrix energy parameter	E_P_ (eV)	21.5	18.7	27.0
Electron effective mass	m_e_ (m_o_)	0.023	0.140	0.039
Luttinger parameters	γ_1_	20.0	5.18	13.40
	γ_2_	8.5	1.19	4.70
	γ_3_	9.2	1.97	6.00
Lattice constant	a (nm)	0.60583	0.61355	0.60959
Elastic stiffness constant	C_11_ (10^11^ dyn·cm^−2^)	8.329	8.769	8.842
	C_12_ (10^11^ dyn·cm^−2^)	4.526	4.341	4.026
Hydrostatic deformation potential				
for conduction band	a_c_ (eV)	−5.08	−4.50	−7.50
for valence band	a_v_ (eV)	−1.00	−1.40	−0.80
Shear deformation potential for valence band	b (eV)	−1.80	−1.35	−2.00

## Data Availability

The data that support the findings of this research are available from the corresponding author upon reasonable request.
